# Preliminary Assessment of Tumor-Associated Tissue Eosinophilia (TATE) in Canine Mast Cell Tumors: Prevalence and Prognostic Relevance and Its Association with Neoangiogenesis

**DOI:** 10.3390/ani13020283

**Published:** 2023-01-13

**Authors:** Valentina Galietta, Francesca Parisi, Cristiano Cocumelli, Alessio Pierini, Alessandro Poli, Paola Scaramozzino, Valentina Spallucci, Francesca Millanta

**Affiliations:** 1 Istituto Zooprofilattico Sperimentale del Lazio e Della Toscana “M. Aleandri”, Via Appia Nuova 1411, 00178 Roma, Italy; 2 Department of Veterinary Sciences, University of Pisa, Viale delle Piagge n. 2, 56124 Pisa, Italy; 3 Agenzia Nazionale per i Servizi Sanitari Regionali, Via Piemonte 60, 00187 Roma, Italy

**Keywords:** dog, eosinophil granulocytes, mast cell tumor, neoangiogenesis, prognosis, tumor associated tissue eosinophilia (TATE), VEGF

## Abstract

**Simple Summary:**

Mast cell tumor (MCT) is the most common malignant skin tumor in dogs. In order to gain more information on the prognostic markers of MCT, the role of the eosinophil granulocytes infiltrates was investigated and assessed by the evaluation of tumor-associated tissue eosinophilia (TATE) in 87 canine cutaneous MCTs. In human medicine, high TATE are often described in highly angiogenic tumors: we therefore assessed the vascular endothelial growth factor (VEGF) expression in neoplastic mast cells. TATE and VEGF expression were compared between themselves, with other variables expressing the biological behavior of the tumor, and with the recurrence. High grades of TATE resulted to be associated with less differentiated tumors, with higher recurrence rates, and with aberrant expression of KIT. This is the first evaluation of the association between TATE and the biologic behavior of MCTs. This study suggests that TATE investigation could be an important source of information for this tumor and for other neoplasms.

**Abstract:**

Mast cell tumor (MCT) is the most common malignant skin tumor in dogs. In order to gain more information on the prognostic markers in MCT, the role of the eosinophil granulocytes infiltrates was investigated and assessed by the evaluation of tumor-associated tissue eosinophilia (TATE) in 87 canine cutaneous MCTs. In human medicine, high TATE are often described in highly angiogenic tumors: we therefore assessed the vascular endothelial growth factor (VEGF) expression in neoplastic mast cells. TATE and VEGF expression were compared between themselves, with histological grading, immunohistochemical expression of KIT and Ki-67, and with the recurrence. We found a statistically significant correlation between TATE and Patnaik grading (*p* = 0.041), Kiupel grading (*p* = 0.022), immunohistochemical KIT expression (*p* = 0.015), and tumor recurrence (*p* = 0.000). No associations were observed with Ki-67 and VEGF expression. This is the first evaluation of TATE and its prognostic value in canine MCTs in veterinary oncology. This study suggest that this investigation could be an important source of information for this tumor and for other neoplasms.

## 1. Introduction

Mast cell tumor (MCT) is considered the most frequent malignant skin tumor in dogs [1], with an incidence of approximately 20% [2]. Cutaneous MCTs appear as non-capsulated masses, solitary or multiple, with dermal localization and/or infiltration of the subcutaneous tissue [3]. Although histological grading has long been considered the main prognostic criterion, recent studies revealed that it is not sufficient to predict the biological behavior of MCTs, but also the support of other prognostic factors is required, such as the expression of KIT and Ki-67 [2]. Furthermore, the tumor microenvironment (TME) has assumed an increasing role in oncology, thus suggesting that its investigation could be an important source of information. Particularly, among the morphological features of different kind of tumors, the eosinophils infiltration has drawn increasing interest. Nowadays, it is clear that eosinophilic infiltration in tumors is an essential component of the TME that influences prognosis and therapeutic response. To describe and emphasize the biological and possibly clinical importance played by eosinophils in different types of cancers, Lowe et al. (1981) [4] proposed the term tumor-associated tissue eosinophilia (TATE). Eosinophils are attracted to the tumor site by cytokines and chemokines, produced and released by different cell types, such as necrotic tumor cells, Th2 lymphocytes, and mast cells [5,6,7]. Once activated, eosinophils are able to synthesize and release a wide variety of biologically active mediators that can have positive or negative effects on various target cells. Reports in the literature about TATE are often contradictory. Some authors proposed an anticancer activity [8,9,10,11] and improvement of survival in patients with TATE [11,12,13]. In contrast, some studies suggested that the detection of eosinophils within a tumor is a negative histological prognostic marker, associated with a more aggressive behavior [14,15,16,17,18]. In particular, they suggest that eosinophils recruited in the tumor site are able to promote angiogenesis and thus tumor growth. Indeed, these cells may directly release proangiogenic molecules, such as vascular endothelial growth factor (VEGF), and also induce the production of this factor by mast cells [19]. VEGF is a heparin-binding protein with an important proangiogenic activity, involved in controlling vascular permeability, and with a mitogenic and an antiapoptotic effect on endothelial cells. VEGF is not only involved in physiological but also in pathological angiogenesis, acting as a regulator or potential autocrine growth factor for neoplastic cells, such as mast cells [20,21,22,23,24].

Taking these data into consideration, our work aims to investigate and quantify TATE, its association with markers of malignancy, angiogenesis, and prognosis in MCTs and with its involvement with the risk of recurrence.

## 2. Materials and Methods

### 2.1. Animals and Tissue Samples

A total of 87 archived formalin-fixed, paraffin-embedded (FFPE) cases of canine cutaneous MCTs were retrieved from the Tumor Registry of the Department of Veterinary Sciences of the University of Pisa. All samples came from solitary nonulcerated MCTs fully excised with clear margins [25], belonged to dogs without any other treatments (e.g., chemotherapy, radiotherapy), and were submitted for histopathology evaluations from 2009 to 2015. For each case, breed, age, sex, and anatomic location of the tumors were recorded, follow-up data on subsequent local and distant tumor recurrence were collected from referring veterinarians.

### 2.2. Histopathology

A 4 μm thick section was cut from each FFPE block and stained with hematoxylin and eosin (HE) for routine histopathology examination. For each MCT, histological grading was assessed according to both the Patnaik et al. (1984) [26] and Kiupel et al. [27] classifications. Histological safety margins were assessed following the latest guidelines proposed by Haine et al. [25], which defined incomplete histological margins as the presence of neoplastic cells within ≤1 mm from the margins. Additionally, TATE was obtained by counting the mean number of eosinophils in 10 consecutive high-power fields (HPF, 2.37 mm^2^), in the hotspot, avoiding necrotic and hemorrhagic areas. The samples were then scored according to the classification provided by Goldsmith et al. (1992) [28]:

- Score 0: none to 2 eosinophils;
- Score 1+: presence of 2–10 eosinophils;
- Score 2+: eosinophils ranging from 10 to 20 eosinophils;
- Score 3+: from 20 to 30 eosinophils;
- Score 4+: more than 30 eosinophils.

### 2.3. Immunohistochemistry

Immunohistochemistry (IHC) investigation was carried out on additional 4 μm thick sections to evaluate the expression of Ki-67, KIT, and VEGF in mast cells, using the streptavidin-biotin peroxidase method.

Briefly, after deparaffinization in xylene and rehydration in graded alcohol according to routine procedures, the endogenous peroxidase activity was blocked by incubating samples with the Bloxall Blocking solution (SP-600, Vector, Burlingame, CA, USA) for 10 min. After microwave cycling to unmask the antigens (three exposures, 5 min each, 750 W in citrate buffer pH 6), nonspecific bindings were blocked by incubation with Ultra V Block (Thermo Fisher Scientific, Fremont, CA, USA) for 5 min. Sections were then incubated over night with a monoclonal mouse antihuman Ki-67 antigen (dilution 1:100, clone MIB-1, Dako, Glostrup, Denmark), a rabbit polyclonal antihuman CD117/KIT (dilution 1:300, Dako, Glostrup, Denmark), and a polyclonal rabbit anti-VEGF (dilution 1:200, A-20, Sanra Cruz Biotechnology, Dallas, TX, USA). Incubation with the primary antibodies was followed the next day by incubation with the Biotinylated antibody universal, Anti-mouse / rabbit IgG, made in Horse (Vector Labs, Inc., Burlingame, CA, USA) for 20 min and incubation with streptavidin–biotin–peroxidase complex (Horseradish Peroxidase Streptavidin, Vector Labs, Burlingame, CA, USA) for twenty min. Colorimetric reaction was revealed with chromogen diaminobenzidine (Impact DAB, Vector Labs, Burlingame, CA, USA). Finally, slides were counterstained with hematoxylin and observed under a light microscope. A canine lymph node and the epidermis as internal control for Ki-67 were used, while for c-KIT and VEGF, a MCT and a canine mammary tumor known to express the marker were employed, respectively. Negative controls were performed by replacing the primary antibody with species-matched unrelated mouse IgG1 isotype control monoclonal antibody (clone MA5-14453, Thermo Fisher Scientific, Fremont, CA, USA) and a rabbit polyclonal anti-toxoplasma antibody (Figure S1).

Samples incubated with Ki-67 were considered positive when the nuclei of mitotic cells showed immunohistochemical staining. Ki-67 index was assessed in all samples using a 1 cm^2^ grid reticle (10 × 10 mm) at 400× *g*. The number of positive cells per grid area was assessed upon highly proliferative areas in 5 HPF and subsequently averaged, as previously reported [29].

Evaluation of KIT expression was carried out in all samples according to Kiupel et al. (2004) [30]. Mast cell tumors were classified in three classes on the base of the staining pattern expressed in neoplastic cell: KIT pattern 1, showing membrane-associated staining; KIT pattern 2, showing focal cytoplasmic (i.e., paranuclear) staining; KIT pattern 3, diffuse cytoplasmic staining. Taking into account 100 neoplastic cells in an HPF, MCTs were classified according to the highest staining pattern in at least 10% cells [29].

VEGF expression was assessed in 72 MCTs using a procedure previously described by Lewis et al. (2000) [31]. After scanning the whole tumor section at 40×, five representative nonadjacent and nonoverlapping fields (most of them VEGF-positive) from each tumor were selected. At 400× *g*, the percentage of positive cells per field (at least 100 of the cells evaluated) was considered, and VEGF expression was determined as the median of the percentages from the five counts. The counts were performed by a single pathologist.

### 2.4. Study Design

Since MCTs with score 4+ of TATE (as previously described by Goldsmith et al., 1992) [28] were poorly represented in terms of numerosity, they were combined with 3+ scores MCTs, creating three categories of TATE (modified TATE classification), as follows:

- Score 1+: ≤10 eosinophils;
- Score 2+: >10 and ≤20 eosinophils;
- Score 3+: >20 eosinophils;

Moreover, we categorized the results of the expression of Ki67 and VEGF, since the distribution of values was not normal.

For all samples the degree of modified TATE and VEGF expression were compared between themselves and with the Patnaik and Kiupel grades, with expression of Ki-67 and KIT, and with the tumor recurrence. Finally, we evaluated the association between histological grading and tumor recurrence.

The associations were evaluated through the chi-squared Pearson test statistics (*p*-value < 0.05). We finally calculated the increasing risk (OR) of recurrence for increasing levels of TATE. The analyses were performed using software Stata 12.0 (StataCorp. College Station, TX, USA).

## 3. Results

The mean age of the dogs of our study at the diagnosis was 7.9 ± 2.8, ranging from 2 to 13 years of age. The Boxer was the most represented breed (Table 1a). Regarding gender, dogs were 50 male and 37 females. Thirty-four MCTs arose on trunk, 31 on limbs, 9 on the head, 8 in inguinal-genital region, 3 in the neck, 2 in the tail region (Table 1b).

Data on postsurgical follow-up were available for 63 dogs. Of these animals, 19.5% had tumor recurrence, while the remaining 80.95% did not show any sign of local or distant recurrences.

According to Patnaik grading [26], 12/87 (13.8%) MCTs were classified as grade 1, 62/87 (71.26%) as grade 2, and 13/87 (14.9%) as grade 3. According to the Kiupel grading [27], 67/87 (77%) cases were classified as low-grade MCTs and 20/87 (23%) as high-grade MCTs.

**Figure 1:**
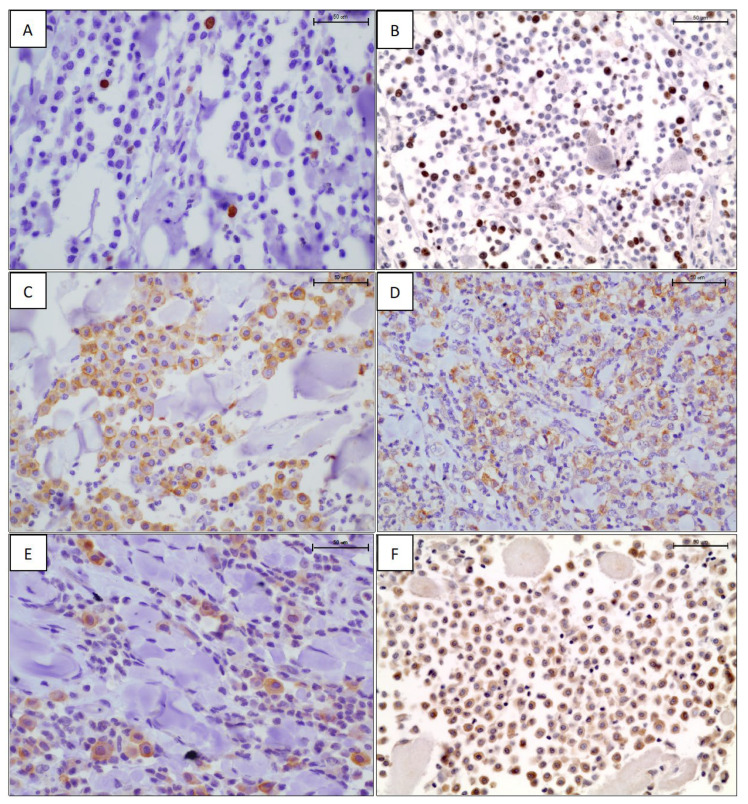
Ki-67 immunostaining: scattered (**A**) and several (**B**), intensely positive nuclei in neoplastic mast cells. Labelled streptavidin biotin (LSAB) method IHC, hematoxylin counterstain, scale bar 50 μm. KIT immunostaining using an anti-CD117 antibody: membranous (**C**), perinuclear (**D**), and cytoplasmic (**E**) staining pattern. LSAB method IHC, hematoxylin counterstain, scale bar 50 μm. (**F**) Diffuse cytoplasmic immunoreactivity to the anti-VEGF antibody. LSAB method IHC, hematoxylin counterstain, scale bar 50 μm.

The results of Ki-67 antigen expression highlighted that 15 mast cell tumors (17.2%) had a Ki-67 index equal to 0 positive nuclei, 22 (25.3%) and 20 (23%) had Ki-67 index of 1 and 2 positive nuclei, respectively, while the remaining had a Ki-67 index ≥3 of positive nuclei, ranging from 3 to 15 (Figure 1A,B, Table 2). Given the low number of positive nuclei in each tumor, for further evaluations, Ki-67 counts were divided into four groups as follows: group 1 = less than 1 stained nucleus; group 2: 1 stained nucleus; group 3: 2 stained nuclei; group 4: ≥3 stained nuclei. They were also divided into three groups as follows: group 1 = no stain; group 2 = 1 or 2 stained nuclei; group 3 = ≥3 stained nuclei. KIT expression showed an intense, membranous staining (KIT pattern 1, Figure 1C) in 17 cases (19.5%), a focal cytoplasmic or paranuclear expression (KIT pattern 2, Figure 1D) in 55 cases (63.2%), and a diffuse cytoplasmic expression (KIT pattern 3, Figure 1E) in the remaining 15 cases (17.3%) (Table 2). Considering the results, we categorized Ki67 both in three and four categories, as reported in Table 2.

VEGF expression was observed in all 72 MCTs tested, with a strong, diffuse to granular, intracytoplasmic pattern of positivity (Figure 1F). The mean percentage of positive cells ranged from 2% to 60%, with a percentage mean of 12.3. Slightly more than half of MCTs had a VEGF expression rate of ≤5% (34/72, 54%) (Table 2). For statical analysis, we categorized the expression of VEGF both in tertiles and using the 5% of IHC expression as cut-off, thus obtaining numerously similar sample groups (Table 2).

From the analysis of modified TATE classification, we observed a significant association with Patnaik grading (*p* = 0.041), Kiupel grading (*p* = 0.022), KIT expression (*p* = 0.015), and tumor recurrence (*p* = 0.000) (Table 3). In contrast, there was no statistically significant association between TATE and expression of Ki-67 and VEGF (*p* > 0.05). Analyzing the VEGF results, we observed a statistically significant association between KIT and VEGF (*p* = 0.033), divided into three categories, and a significant association between Kiupel grading and VEGF in two categories (*p* = 0.019) (Table 3). Finally, considering tumor recurrence, we found a significant association with Patnaik grading (*p* = 0.022) (Table 3).

The estimated risk of recurrence (OR) for the third grade of TATE with respect to the first grade was 3.2 (CI95% = 0.61–16.67).

**Figure 2:**
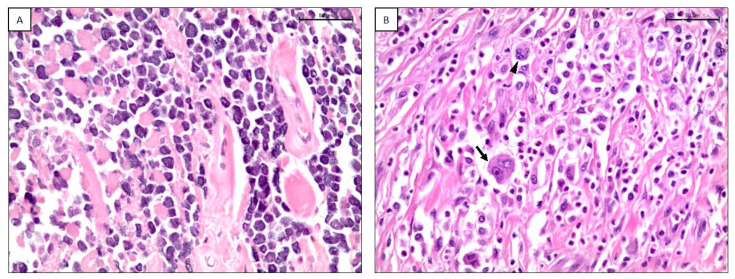
(**A**) Canine cutaneous mast cell tumor with a TATE score of 1+. H-E, scale bar 50 μm. (**B**) Canine cutaneous mast cell tumor with a TATE score of 3+. Arrow: binucleation; arrowhead: multinucleation. H-E, scale bar 50 μm.

According to the modified-TATE classification score, 22/87 (25.3%) MCTs were classified as score 1+ (Figure 2A), 42/87 (48.3%) as score 2+, 23/87 (26.4%) as score 3+ (Figure 2B) (Table 2).

## 4. Discussion

MCTs may have highly variable biological behavior. Histological grading has long been considered the main prognostic criterion, but it is now evident that other factors are needed to predict the biological behavior of MCTs. None of the acknowledged markers have a completely reliable prognostic value. Therefore, the greatest predictive benefit comes from the combined use of different markers [32]. For this reason, many research works, including the present study, are focused on the investigation of new prognostic factors that can predict more accurately the outcome of MCTs.

The choice to restrict our study to solitary lesions excised with clear margins finds a rationale in the attempt to control the variability of the prognosis. Indeed, some authors [33] suggested that the presence of multiple MCTs may negatively influence the prognosis, while, on the contrary, others [34] suggested that overall survival was not influenced by it. Moreover, incisional biopsies have not been entered in the study because they may not be faithfully representative of the entire lesion, whose comprehensive analysis is required for the Patnaik histological grading [1].

In our study, Boxers and Retrievers and older dogs were confirmed among the most affected subjects, with a medium age comparable to that reported in previous studies, and no clear gender predisposition was highlighted [1,35]. The trunk was confirmed as the most affected region, followed by the limbs, the head, and the neck, as already observed [36]. On the contrary, we did not find a large number of inguinal-genital MCTs that were previously reported as frequently as trunk MCTs [37,38]. The reason for this discrepancy can be found in the type of samples: our study focuses only on cutaneous tumors, while most of the other works in the literature combine cutaneous and subcutaneous mast cell tumors.

The assessment of histological grading in MCTs remains the key point for predicting prognosis. The Patnaik classification was reported to be more sensitive, whereas the Kiupel one seems to be more specific in detecting aggressive diseases [39]. In line with the latest recommendations of the second Consensus Meeting on the Diagnosis, Prognosis, and Treatment of Canine Cutaneous and Subcutaneous Mast Cell Tumors organized by the Brazilian Association of Veterinary Oncology (ABROVET) [1] and of the American College of Veterinary Pathologists and the Veterinary Cancer Society [40], our study applied both of the classification schemes [26,27]. Comparing the two grading systems, we observed that all Patnaik grade 1 cases, most Patnaik grade 2 cases (85.5%), and two grade 3 cases (15.4%) were included in Kiupel low grades. Kiupel high-grade MCTs, on the other hand, included the remaining Patnaik grade 2 and most Patnaik grade 3 (84.6%). This indicates a correspondence between the two classification systems, especially between Patnaik grades 1 and 3 and Kiupel low and high grade, respectively. Patnaik grade 2 cases were instead distributed among the Kiupel grades, as already observed by previous authors using both classification schemes [39,41,42,43].

Latest studies demonstrated that immunohistochemical markers are required to better characterize the biological behavior of all MCTs. We decided to evaluate expression of Ki-67 and KIT, as already consolidated and more reliable prognostic factors [44]. The results of the Ki-67 index pointed out that most cases with a low number of positive nuclei were classified as Kiupel low grade, while most of those with a high number of positive nuclei at Ki67 were Kiupel high grade, confirming the value of this parameter as a prognostic factor [44]. We found an association between KIT expression pattern and tumor recurrence rates. This result is in line with previous studies. KIT cytoplasmic immunoreactivity was correlated with a reduced survival [30,45], a higher histological grade, and an increase in cell proliferation [46]. Particularly, Kiupel et al. [30] associated KIT patterns 2 and 3 with a higher local recurrence rate and a higher mortality rate in their study.

All the MCTs tested positive for VEGF protein, and although not statistically significant, tumors with more conspicuous malignant features showed a trend towards a higher expression. In mouse and human mast cells, the release of VEGF is already described [47], and in other studies, its role in the regulation of angiogenesis confirmed that mast-cell-derived VEGF is involved as well [48,49,50].

In humans, it has been proved that the VEGF released by mast cells, after being activated by several molecules and mechanisms, is strictly involved in tumor development [51,52]. Rebuzzi et al. [53] succeeded in demonstrating that primary neoplastic mast cells in dogs and a canine MCTs cell line express VEGF protein at the mRNA and protein level. Although the angiogenic power of mast cells is known, there are conflicting results in the literature on its prognostic value. One study indicated that VEGF expression was predictive of poor prognosis [1]. On the contrary, other authors highlighted that VEGF had no value as a biomarker for malignancy and was not useful to predict clinical outcome [53,54]. Actually, Amorim et al. [54] suggested that MCTs probably expressed VEGF because their neoplastic cells retained the capability to release this proangiogenic factor typical of their physiologic status, although both in their study and in that of Rebuzzi et al. [53], VEGF appeared not to act as an autocrine growth regulator. Given these previous considerations and the results of our study, it is likely that angiogenetic function could be accomplished also by other mediator factors in MCTs, and that further studies are needed to provide additional data also on the paracrine regulation of VEGF production.

Nowadays, the analysis of TME is at the forefront of oncology since tumor infiltrating cells or immune cells were reported to influence the development and the progression of several tumoral diseases. Hence, for this reason, we focused our attention on eosinophils. Eosinophils are immunomodulatory, cytotoxic cells representing 1–3% of leukocytes that have been reported in various organs associated with the pathogenesis and prognosis of various pathologies, such as allergic phenomena, asthma, vasculitis, gastrointestinal disorders, and parasitosis [55]. The term TATE was suggested at first by Lowe et al. in 1981 [4] to indicate the presence of eosinophils in tumor stroma recruited under the influence of chemicals or other immune cells, in the absence of any ulceration or necrosis [56,57]. TATE has been documented in several neoplasms in human medicine, such as oral squamous cell carcinoma (SCC) [58], solid tumors, bladder cancer [18], larynx SCC [59], esophageal SCC [60], colorectal cancer [61], cervical carcinoma [62], gastric adenocarcinoma [63], nasopharyngeal carcinoma [64], Hodgkin lymphoma [65], and penile cancer [13]. The presence of eosinophils in MCT is due to mast-cell-released eosinophil chemoattractant factors (such as histamine, stem cell factor, and interleukin 5) [66]; however, there are no studies testing the potential prognostic power of the degree of eosinophils infiltrates in MCTs.

To date, and to the authors knowledge, there are no reports about TATE with tumors in veterinary medicine.

Due to this lack of information, we first quantified eosinophils in canine MCTs using the score proposed by Goldschmidt et al., 1992 [28], but then we modified it in a three-point one, since the latest group was not represented in the population of MCTs analyzed. Whether this eventuality is linked to the species difference or to the different neoplasms analyzed, it should be clarified. In our study, a high number of intratumor eosinophils (modified TATE score 3+) was significantly associated with less differentiated tumors following both the Patnaik and Kiupel histological classification, with aberrant KIT expression (KIT pattern 2 and 3). The estimated risk of recurrence (OR) for the third grade of TATE with respect to the first grade was >1, but it was not significant at 95% CL, probably due to the small number of observations. Nevertheless, this evidence suggested that a high score of tissue eosinophilia should be considered as a negative prognostic marker for canine MCTs. This finding also highlights that not only do mast cells modulate eosinophils activation, but that the reverse influence is also possible, with mast cells and eosinophils having a bidirectional interaction in TME [19,67]. Moreover, it seems that specific chemotactic molecules of cancer, known as eosinophil chemotactic factor of anaphylaxis, (ECF-Ca), different from the chemotactic factor of anaphylaxis eosinophil chemotactic factor of anaphylaxis (ECF-A) exist [68].

Previous studies in humans suggested that tissue eosinophilia in different tumors may be associated either with good or poor patient prognosis [57]. Some authors highlighted that tissue eosinophils hesitated into an antitumor role and, therefore, resulted in better prognosis, due to the secretion of cytotoxic mediators, such as the main basic protein (MBP), the eosinophilic cationic protein (CEP), and the eosinophilic peroxides (EP) acting as tumoricidal agents [16]. On the other hand, other researchers [69] reported that ECP could degrade muscle and cytoskeletal proteins associated with cell membrane, thus contributing to muscle fiber degradation and tumor invasion. Additional studies suggested that tissue eosinophils could promote neoangiogenesis and tumor development through the releasing of proangiogenic factors, such as VEGF, remodeling factors, such as fibroblast growth factor (b-FGF), GM-CSF, TGF-b, and MMP-9, but also IL-6, IL-8, and platelet-derived growth factor (PDGF) [6,69,70,71,72]. Particularly, Alrawi et al. [14] observed that an elevated eosinophilic count in SCC of the digestive tract was associated with tumor invasion and, therefore, predictive of an aggressive tumor form. Rakesh et al. [73] found that relapse of oral SCC was significantly associated with a great number of TATE. Choudhary et al. [57] hypothesized that the variable outcome among different studies could be also related to some intrinsic factors, such as different clinical and pathological stages of neoplasms, different histological grades, and the presence or absence of metastases. They have also opened a debate on the methodology by which eosinophilia was assessed by various researchers, claiming a need for standardization of the different counting methodologies, which could seriously affect the results of the research output. Particularly, they suggested to count per high power field rather than per mm^2^, because the latter requires a morphometric software which is not available in every pathology facility. Finally, TATE investigation requires no additional cost and time for staining or pathological processing, hence further investigation on this topic could also be facilitated by low costs.

Bearing in mind that a better clinical and therapeutic management of MCTs, as well as a more accurate prediction of the outcome, can be achieved through the use of an accurate clinical staging [74,75] and a combination of multiple prognostic markers, we believe that, on the basis of the results and the considerations of this study, TATE should be taken into consideration as a prognostic factor in MCT. On the basis of our results, although preliminary, it would be worthwhile trying to apply this TATE classification scheme to cytological samples, with far more immediate implications in the clinical management of MCTs. Moreover, due to their involvement in TME and their interaction with several immune molecules and cells influencing tumor development and the low cost of the procedures, further studies to elucidate the role of eosinophils in neoplasms of other origins should be also encouraged.

## 5. Conclusions

Based on our knowledge, this work has evaluated for the first time in veterinary oncology the prognostic role of eosinophilic infiltration assessed by TATE quantification in canine mast cell tumors. Despite conflicting data in the literature, this preliminary study highlights the usefulness of evaluating the prognostic importance of TATE in canine MCT and other neoplasms, to further understand the role of TME in the development of neoplasms and, therefore, the implementation of new therapeutic strategies.

## Figures and Tables

**Table 1:** Details of the breeds with MCTs selected for the study (a), and list of the tumor sites (b).

**(a)—Breeds**	
Boxer	19
Mixed breed	15
Labrador Retriever	10
English Setter	6
Argentine Dogo	5
Pug	5
Jack Russell	3
Corsican	3
Beagle	2
German Hound	2
Bull Mastiff	2
French Bulldogs	2
Golden Retrievers	2
Scottish Shepherd Dogs	2
Springer Spaniel	2
Yorkshire terrier	2
Single dogs grouped (Border collie, Chihuahua, Pincher, Rottweiler, Italian Hound)	5
**(b)—Tumor Site**	
Trunk	34
Limbs	31
Head	9
Inguinal-genital	8
Neck	3
Tail	2

**Table 2:** Summary of the results for the different variables analyzed and their categories/classification. Modified TATE is expressed in three scores; Ki-67 values are both divided in 4 and in 3 groups; KIT in three patterns of expression; VEGF expression is reported in tertiles and with a cut-off value of 5%.

Variable	Categories/Classification	*n*° and % of MCTs		Total *n*° of MCTs
Modified TATE				
	Score 1	22	25.3%	87
	Score 2	42	48.3%	
	Score 3	23	26.4%	
Ki-67 expression				
	4 groups			
	1	15	17.2%	87
	2	22	25.3%	
	3	20	23.0%	
	4	30	34.5%	
	3 groups			
	1	15	17.2%	87
	2	42	48.3%	
	3	30	34.5%	
KIT expression				
	Pattern 1	17	19.5%	87
	Pattern 2	55	63.2%	
	Pattern 3	15	17.2%	
VEGF expression				
	tertiles			
	1° tertiles	52	72.2%	72
	2° tertiles	15	20,8%	
	3° tertiles	5	6.9%	
	cut-off value			
	≤5%	41	56.9%	72
	>5	31	43.1%	
Patnaik grading				
	1	12	13.8%	87
	2	62	71.3%	
	3	13	14.9%	
Kiupel grading				
	Low grade	67	77.0%	87
	High grade	20	23.0%	
Recurrence				
	Yes	12	19.0%	63
	No	51	81.0%	

**Table 3:** Association between immunohistochemical markers, histological grading, recurrence, VEGF expression, and modified TATE.

	Modified TATE	VEGF (2 Categories)	VEGF (3 Categories)
Variable	*p*-Value	*p*-Value	*p*-Value
KIT expression	0.015	0.185	0.033
Ki-67 expression (3 groups)	0.786	0.932	0.704
Ki-67 expression (4 groups)	0.809	0.986	0.588
VEGF (cut-off value 5%)	0.250	//	//
VEGF (tertiles)	0.445	//	//
Patnaik grading	0.041	0.177	0.986
Kiupel grading	0.022	0.019	0.896
Recurrence	0.000	0.104	0.433

## Data Availability

The data presented in this study are available in this article.

## Supplementary Materials

The following are available online at https://www.mdpi.com/article/10.3390/ani13020283/s1, Figure S1: (A) Canine lymph node, positive control for the anti-Ki67 antibody (mouse anti-human Ki-67, clone MIB-1, Dako, Glostrup, Denmark), inset: an immunolabelled mitosis (yellow arrow). IHC, EE counterstaining, bar = 100 µm (B) Canine MCT, negative control for the anti-Ki67 antibody (mouse anti-human Ki-67 antigen, clone MIB-1, Dako, Glostrup, Denmark), IHC, EE counterstaining, bar = 100 µm (C) Canine MCT, positive control for the anti- CD117/KIT antibody (rabbit polyclonal anti-human CD117/KIT Dako, Glostrup, Denmark), IHC, EE counterstaining, bar = 50 µm (D) Canine MCT, negative control for the anti-CD117/KIT antibody (rabbit polyclonal anti-human CD117/KIT Dako, Glostrup, Denmark), IHC, EE counterstaining, bar = 100 µm E) Canine mammary gland tumor, positive control for the anti-VEGF antibody (rabbit polyclonal anti-VEGF, A-20, Santa Cruz Biotechnology, Texas, USA). IHC, EE counterstaining, bar = 50 µm F) Canine MCT, negative control for the anti-VEGF antibody (A-20, Santa Cruz Biotechnology, Texas, USA), IHC, EE counterstaining, bar = 100 µm.
