# Supplementary material for: Preliminary Assessment of Tumor-Associated Tissue Eosinophilia (TATE) in Canine Mast Cell Tumors: Prevalence and Prognostic Relevance and Its Association with Neoangiogenesis
Source: Animals (Basel). 2023 Jan 13;13(2):283. doi: 10.3390/ani13020283 (PMC9855198; doi:10.3390/ani13020283)
Supplement: Supplementary file 1 [file animals-13-00283-s001.zip › animals-2103662-supplementary.pdf]

animals

MDPI

Supplementary Materilas

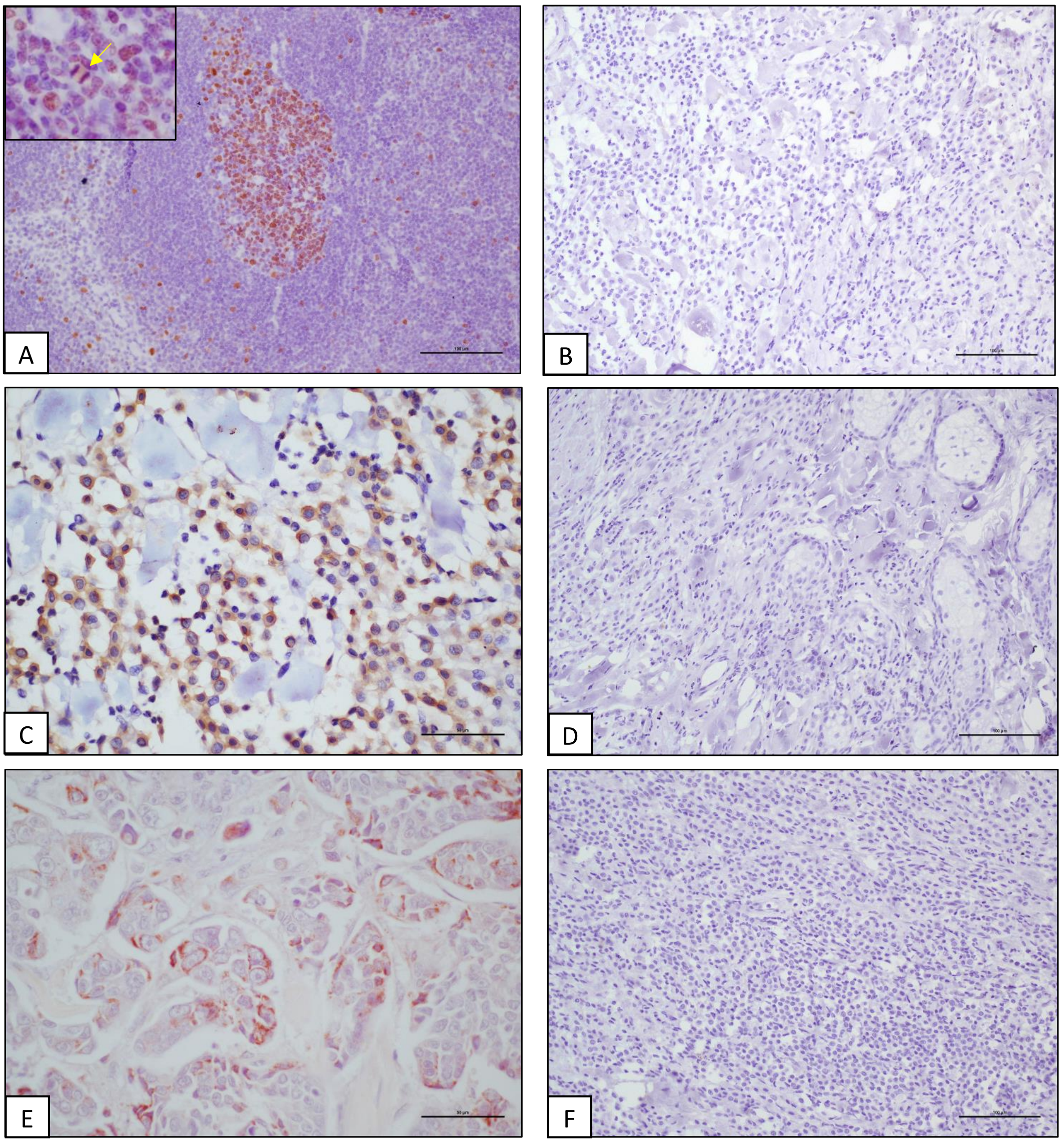

Figure S1. (**A**) Canine lymph node, positive control for the anti-Ki67 antibody (mouse anti-human Ki-67, clone MIB-1, Dako, Glostrup, Denmark), inset: an immunolabelled mitosis (yellow arrow). IHC, EE counterstaining, bar= 100 μm (**B**) Canine MCT, negative control for the anti-Ki67 antibody (mouse anti-human Ki-67 antigen, clone MIB-1, Dako, Glostrup, Denmark), IHC, EE counterstaining, bar= 100 μm (**C**) Canine MCT, positive control for the anti- CD117/KIT antibody (rabbit polyclonal anti-human CD117/KIT Dako, Glostrup, Denmark), IHC, EE counterstaining, bar= 50 μm (**D**) Canine MCT, negative control for the anti-CD117/KIT antibody (rabbit polyclonal anti-human CD117/KIT Dako, Glostrup, Denmark), IHC, EE counterstaining, bar= 100 μm (**E**) Canine mammary gland tumor, positive control for the anti-VEGF antibody (rabbit polyclonal anti-VEGF, A-20, Santa Cruz Biotechnology, Texas, USA). IHC, EE counterstaining, bar= 50 μm (**F**) Canine MCT, negative control for the anti-VEGF antibody (A-20, Santa Cruz Biotechnology, Texas, USA), IHC, EE counterstaining, bar= 100 μm.
